# Retraction: Synthesis and characterization of an Fe-MOF@Fe_3_O_4_ nanocatalyst and its application as an organic nanocatalyst for one-pot synthesis of dihydropyrano[2,3-c]chromenes

**DOI:** 10.3389/fchem.2026.1959100

**Published:** 2026-08-17

**Authors:** Frontiers Editorial Office

The journal retracts the 04 January 2023 article cited above.

Following publication, concerns were raised about the integrity of the images in the published figures. The authors failed to provide a satisfactory explanation during the investigation, which was conducted in accordance with Frontiers' policies.

This retraction was approved by the Chief Executive Editor of Frontiers. The authors do not agree to this retraction.

